# *Slco*2*a1* deficiency exacerbates experimental colitis via inflammasome activation in macrophages: a possible mechanism of chronic enteropathy associated with *SLCO2A1* gene

**DOI:** 10.1038/s41598-020-61775-9

**Published:** 2020-03-17

**Authors:** Rieko Nakata, Yoshinobu Nakamura, Shuhei Hosomi, Hiroaki Okuda, Yu Nishida, Naoko Sugita, Shigehiro Itani, Yuji Nadatani, Koji Otani, Fumio Tanaka, Noriko Kamata, Koichi Taira, Yasuaki Nagami, Tetsuya Tanigawa, Toshio Watanabe, Hirokazu Yamagami, Takeo Nakanishi, Yasuhiro Fujiwara

**Affiliations:** 1 Department of Gastroenterology, Osaka City University Graduate School of Medicine, Osaka, Japan; 2 Department of Pharmacy, Shiga University of Medical Science Hospital, Shiga, Japan; 3 Faculty of Pharmacy, Takasaki University of Health and Welfare, Gunma, Japan

**Keywords:** Intestinal diseases, Ulcers, Mechanisms of disease, Inflammation, Mucosal immunology

## Abstract

Loss-of-function mutations in the solute carrier organic anion transporter family, member 2a1 gene (*SLCO2A1*), which encodes a prostaglandin (PG) transporter, have been identified as causes of chronic nonspecific multiple ulcers in the small intestine; however, the underlying mechanisms have not been revealed. We, therefore, evaluated the effects of systemic knockout of *Slco2a1* (*Slco2a1*^−/−^) and conditional knockout in intestinal epithelial cells (*Slco2a1*^*ΔIEC*^) and macrophages (*Slco2a1*^*ΔMP*^) in mice with dextran sodium sulphate (DSS)-induced acute colitis. *Slco2a*^−/−^ mice were more susceptible to DSS-induced colitis than wild-type (WT) mice, but did not spontaneously develop enteritis or colitis. The nucleotide-binding domain, leucine-rich repeats containing family, pyrin domain-containing-3 (NLRP3) inflammasome was more strongly upregulated in colon tissues of *Slco2a*^−/−^ mice administered DSS and in macrophages isolated from *Slco2a1*^−/−^ mice than in the WT counterparts. *Slco2a1*^*ΔMP*^, but not *Slco2a1*^*ΔIEC*^ mice, were more susceptible to DSS-induced colitis than WT mice, partly phenocopying *Slco2a*^−/−^ mice. Concentrations of PGE_2_ in colon tissues and macrophages from *Slco2a1*^−/−^ mice were significantly higher than those of WT mice. Blockade of inflammasome activation suppressed the exacerbation of colitis. These results indicated that *Slco2a1*-deficiency increases the PGE_2_ concentration, resulting in NLRP3 inflammasome activation in macrophages, thus exacerbating intestinal inflammation.

## Introduction

Prostaglandin (PG) E_2_ is the most physiologically abundant eicosanoid biosynthesized from arachidonic acid by cyclooxygenase (COX). PGE_2_ has important roles in maintaining gut mucosal homeostasis^1,2^, but also is an essential mediator of the immune response and inflammation in various inflammatory diseases^3^. Indeed, small intestinal ulcers can be induced by nonsteroidal anti-inflammatory drugs (NSAIDs) treatment through suppressing PGE_2_ synthesis by inhibition of COX. PGE_2_ is exported to the extracellular microenvironment by multiple drug resistance-associated protein 4 (MRP4/ABCC4)^4^ and exerts its effects by binding to a family of G protein-coupled receptors consisting of four subtypes: EP1, EP2, EP3, and EP4^5^. Prostaglandin transporter (PGT), encoded by the solute carrier organic anion transporter family, member 2a1 gene (*SLCO2A1*) mediates cellular uptake of PGE_2_^6,7^. PGE_2_ is oxidized intracellularly by 15-ketoprostaglandin dehydrogenase (15-PGDH; encoded by *HPGD*)^8,9^. Thus, PGT plays an important role in PGE_2_ metabolism. Umeno *et al*. recently reported that chronic enteropathy, which is characterized by multiple intractable small-intestinal ulcers with manifestations including anaemia and hypoproteinaemia^10,11^, is caused by autosomal recessive loss-of-function mutations in *SLCO2A1*^12^. PGE_2_ concentrations in patients with this genetic background should theoretically be higher than those in patients without this mutation because of lower PGE_2_ metabolism. In fact, concentrations of PGE major urinary metabolites, which are stable metabolites derived from PGE_2_, in patients with chronic enteropathy, so-called chronic enteropathy associated with *SLCO2A1* gene (CEAS), have been shown to be higher than those in patients with Crohn’s disease^13^. Thus, given the protective effect of PGE_2_ on mucosal injury, PGE_2_ seems to exert contrasting effects on the intestinal mucosa, and the mechanisms underlying enteritis development under *SLCO2A1* deficiency have not been clarified.

In the present study, we evaluated a potential role of SLCO2A1 in intestinal homeostasis using systemic as well as conditional *Slco2a1*-knockout mice. Deletion of *Slco2a1* did not cause alteration of epithelial structure and spontaneous enteritis. However, SLCO2A1 protected against gut inflammation in an experimental colitis model. Further, we report a possible mechanism: increased PGE_2_ because of *Slco2a1* deficiency autocrinally causes inflammasome activation in macrophages, resulting in exacerbation of intestinal inflammation.

## Results

### *Slco2a1* deficiency does not result in spontaneous enterocolitis

**Figure 1:**
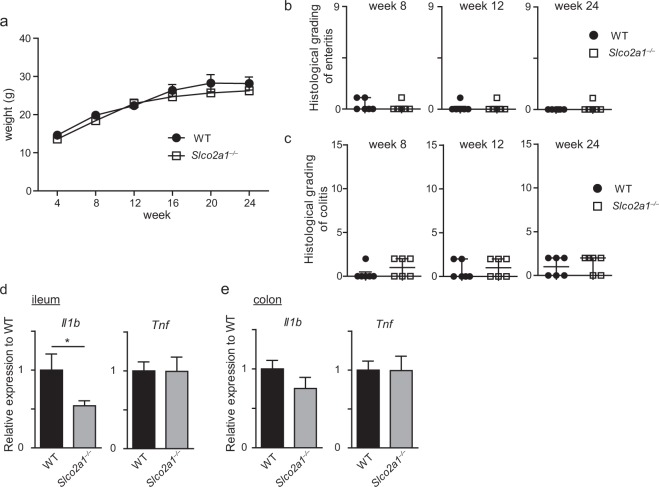
Systemic *Slco2a1* deficiency does not induce spontaneous intestinal inflammation. (**a**) Body weight changes in WT and *Slco2a1*^−/−^ mice during 24 weeks (n = 8–22). (**b**,**c**) Histological scores for mucosal inflammation in the ileum and colon in 8, 12, and 24-week-old WT and *Slco2a1*^−/−^ mice. (**d**,**e**) mRNA expression levels of *Il1b* and *Tnf* in ileum and colon tissues of 8-week-old WT and *Slco2a1*^−/−^ mice as assessed by RT-qPCR. Data represent the mean ± SEM or median and IQR. Statistical significance was calculated using Student’s *t*-test, Welch’s *t*-test, or Mann-Whitney test (*P < 0.05).

To investigate the effect of *Slco2a1* deficiency on the intestine, germline *Slco2a1-*knockout (*Slco2a1*^−/−^) mice^14^ were evaluated for basal phenotype in comparison with wild-type (WT) mice. There were no significant differences in body weight between *Slco2a1*^−/−^ and WT mice at any time point evaluated (Fig. 1a). *Slco2a1*^−/−^ mice did not display any sign of enteritis as assessed by histological examination of ilea and colons at 8, 12, and 24 weeks of age (Fig. 1b,c, and Supplementary Fig. S1a,b). mRNA levels of *Il1b* and *Tnf*, which are pivotal inflammatory cytokines^15^, in the colon were not affected by *Slco2a1* knockout, although *Il1b* mRNA expression in the ileum of *Slco2a1*^−/−^ mice was decreased (Fig. 1d,e). These results demonstrated that *Slco2a1* deficiency *per se* does not induce intestinal inflammation. *Slco2a1* deficiency also did not affect the numbers of goblet cells and Paneth cells, which secrete mucins and antibacterial contents for mucosal barrier function^16,17^ (Supplementary Fig. S2a,b).

### *Slco2a1*^−/−^ mice are more susceptible to DSS-induced colitis

**Figure 2:**
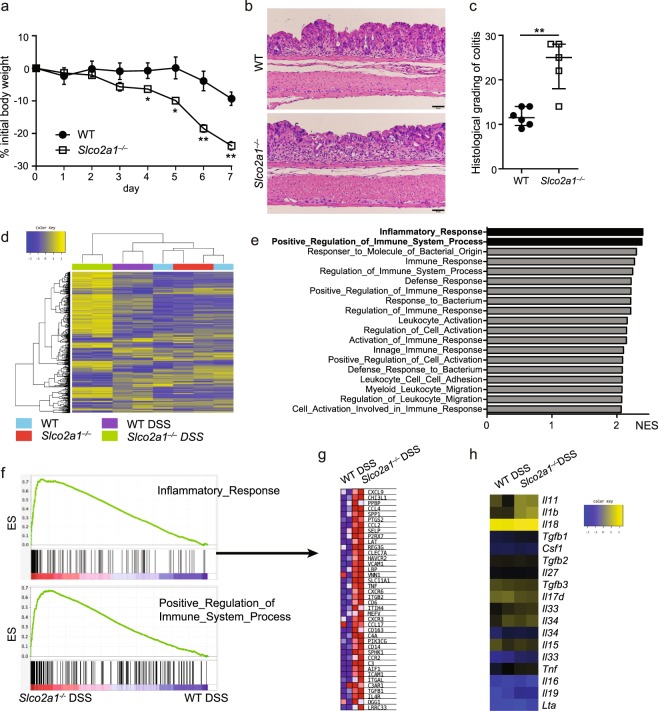
Systemic *Slco2a1* deficiency increases susceptibility to dextran sodium sulphate (DSS)-induced colitis. (**a**) Changes in body weight during the experimental period. WT (n = 6) and *Slco2a1*^−/−^ (n = 6) mice were administered 3.5% DSS for 7 days. (**b**,**c**) Representative H&E-stained colon sections (scale bars: 50 µm) and histological colitis scores for WT and *Slco2a1*^−/−^ mice on experimental day 7. (**d**–**h**) Microarray analysis of mRNA expression in the colon. (**d**) Heatmap showing whole-gene expression profiles for WT and *Slco2a1*^−/−^ mice treated with or without DSS for 7 days. (**e**) Top-20 enriched GO terms for genes upregulated in *Slco2a1*^−/−^ vs. WT mice treated with DSS as revealed by GSEA. (**f**) GSEA plots of immune response and positive regulation of immune system processes. (**g**) Heatmap showing the relative expression levels (red = high, blue = low) of the 40 most strongly upregulated genes involved in the inflammatory response in *Slco2a1*^−/−^ vs. WT mice with colitis. (**h**) Heatmap of cytokine expression in WT and *Slco2a1*^−/−^ mice with colitis (**h**). Data represent the mean ± SEM or median and IQR. Statistical significance was calculated by Student’s *t*-test, Welch’s *t*-test, or Mann-Whitney test (*P < 0.05, **P < 0.01).

To investigate the role of SLCO2A1 in colitis development, we next examined the effects of *Slco2a1* deficiency in experimental colitis models of *Slco2a1*^−/−^ and WT mice established by administering 3.5% DSS in the drinking water for 7 days. *Slco2a1*^−/−^ mice lost significantly more weight than did WT mice upon DSS-induced colitis (Fig. 2a). Histopathological examination of colons revealed more severe lesions, stronger neutrophil and lymphocyte infiltration into the mucosal and submucosal areas, and more extensive loss of crypts in *Slco2a1*^−/−^ mice than in WT mice, resulting in higher histological colitis grades (Fig. 2b,c).

To investigate the mechanisms underlying colitis exacerbation in *Slco2a1*^−/−^ mice further, we analysed gene expression profiles in colon tissues of *Slco2a1*^−/−^ and WT mice in the presence/absence of DSS-induced colitis by microarray analysis (Fig. 2d). Gene set enrichment analysis (GSEA) indicated that the most strongly enriched pathways in *Slco2a1*^−/−^ mice with DSS-induced colitis were related to the inflammatory immune response and leukocyte migration/activation (Fig. 2e,f). Comparison of the expression levels of enriched genes in the inflammatory response (Fig. 2g) and cytokine expression (Fig. 2h) revealed that the expression of inflammasome-related genes, such as *Il1b*, *Il18*, *P2rx7*, and macrophage-related chemokines, such as *Ccl2* and *Ccl4*, was upregulated in *Slco2a1*^−/−^ compared to WT mice administered DSS. The proinflammatory cytokine interleukin (IL)-1β, which is cleaved and activated by inflammasome assembly^18^, and the nucleotide-binding domain, leucine-rich repeats containing family, pyrin domain-containing-3 (NLRP3) inflammasome reportedly play important roles in inflammation in DSS-induced colitis model mice^19^. Inflammasomes activation is involved in other experimental enteritis/colitis models^20,21^ and is related to human inflammatory bowel disease (IBD) via genetic and environmental factors^22,23^.

### IL-1β production induced by inflammasome activation is increased in *Slco2a1*^−/−^ mice

**Figure 3:**
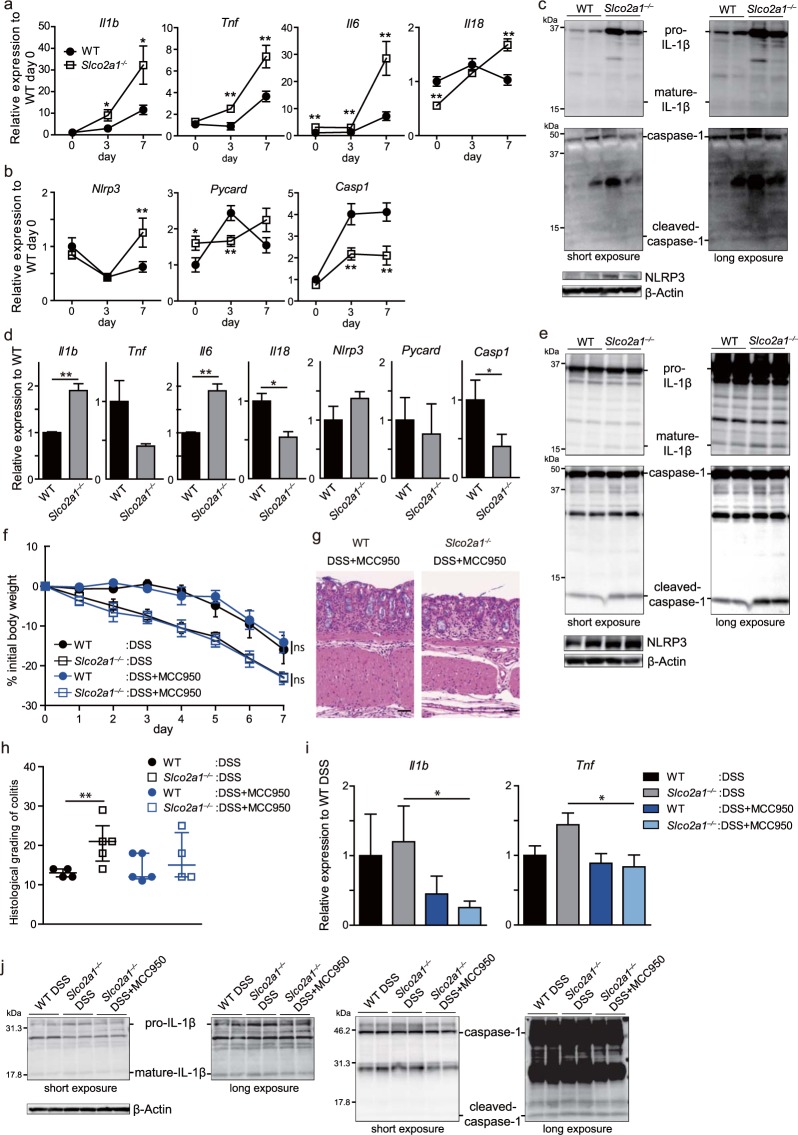
NOD-like receptor protein 3 (NLRP3) inflammasome is activated in *Slco2a1*^−/−^ mice administered dextran sodium sulphate (DSS). (**a**,**b**) mRNA levels of inflammatory cytokines (**a**) and NLRP3 inflammasome-associated molecules (**b**) in colonic tissues on experimental days 0, 3, and 7 as assessed by RT-qPCR. (**c**) Western blot assessment of pro- and mature IL-1β, pro- and cleaved caspase-1, and β-Actin in colonic tissues on experimental day 7. β-Actin was used for normalization. (**d**) mRNA levels of inflammatory cytokines and NLRP3 inflammasome-related genes in LP macrophages isolated from WT and *Slco2a1*^−/−^ mice treated with 3.5% DSS for 3 days. (**e**) BMDMs from WT and *Slco2a1*^−/−^ mice were stimulated with 1 μg/mL of LPS for 4 h and then analysed NLRP3 for inflammasome-related molecules by western blotting. β-Actin was used for normalization. (**f**) Changes in body weights of WT (n = 6) and *Slco2a1*^−/−^ (n = 6) mice intraperitoneally treated with MCC950 (NLRP3 inhibitor) or PBS during 3.5% DSS administration. (**g**,**h**) Representative H&E-stained ileum and colon sections (scale bars: 50 µm) and histological colitis scores. (i) mRNA levels of *Il1b* and *Tnf* in colon tissues from WT and *Slco2a1*^−/−^ mice on experimental day 7. (**j**) Western blot assessment of pro- and mature IL-1β, pro- and cleaved caspase-1, and β-Actin in colonic tissues on experimental day 7. Data represent mean ± SEM or median and IQR. Statistical significance was calculated by Student’s *t*-test, Welch’s *t*-test, or Mann-Whitney test (*P < 0.05, **P < 0.01).

We next focused on the role of the NLRP3 inflammasome in the exacerbated colitis in *Slco2a1*^−/−^ mice. mRNA expression of pro-inflammatory cytokines in colon tissues as assessed by RT-qPCR was increased in *Slco2a1*^−/−^ compared to WT mice on days 3 and 7 of DSS treatment (Fig. 3a)., Although *Casp1* mRNA expression was decreased (Fig. 3b), protein levels of pro- and cleaved IL-1β, cleaved caspase-1 (CASP1), and NLRP3 were also significantly elevated in *Slco2a1*^−/−^ compared to WT mice with colitis (Fig. 3c, Supplementary Fig. S3). These results indicated that NLRP3 inflammasome activation might be associated with the exacerbation of DSS colitis in *Slco2a1*^−/−^ mice. The NLRP3 inflammasome and subsequent secretion of mature IL-1β by macrophages reportedly are a critical mechanism of intestinal inflammation in the DSS-induced acute colitis mouse model^19^. Considering this fact together with the microarray data, we next focused on the inflammasome pathway in intestinal macrophages, and investigated whether inflammasome activation in macrophages could exacerbate the intestinal inflammation. To assess the inflammasome status in macrophages with genetic *Slco2a1* deletion, we isolated colonic lamina propria (LP) macrophages from WT and *Slco2a1*^−/−^ mice with DSS-induced colitis. Consistent with the results *in vivo*, *Il1b* mRNA expression was significantly increased, whereas *Casp1* mRNA expression was significantly decreased in *Slco2a1*^−/−^ mice (Fig. 3d). *Nlrp3* mRNA expression was elevated, but not significantly. In line with the mRNA data for LP macrophages, the protein levels of NLRP3, cleaved caspase-1, and mature IL-1β were significantly increased in bone marrow-derived macrophages (BMDMs) from *Slco2a1*^−/−^ compared to the levels in BMDMs from WT mice (Fig. 3e, Supplementary Fig. S4). Accordingly, although administration of MCC950, a specific small-molecule inhibitor of NLRP3 inflammasome activity^24^, did not affect body weight changes (Fig. 3f), it reduced the severity of colitis in *Slco2a1*^−/−^ mice (Fig. 3g,h), diminished the *Il1b* and *Tnf* mRNA levels (Fig. 3i) with suppressed formation of mature IL-1β and cleaved caspase-1(Fig. 3j, Supplementary Fig. S5), suggesting that the NLRP3 inflammasome contributed to exacerbation of DSS colitis under *Slco2a1* deletion.

### *Slco2a1* deficiency in macrophages, but not intestinal epithelial cells, alters susceptibility to DSS-induced colitis

**Figure 4:**
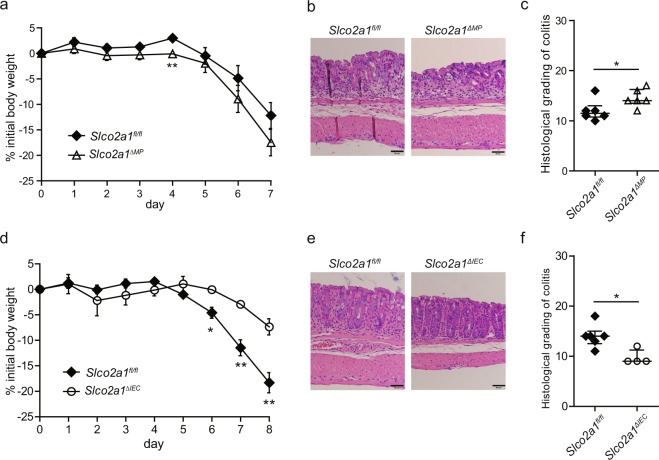
*Slco2a1* deficiency in macrophages alters susceptibility to dextran sodium sulphate (DSS)-induced colitis. (**a**) Changes in body weight during the experimental period. *Slco2a1*^*fl/fl*^ (n = 6) and *Slco2a1*^*ΔMP*^ (n = 6) mice were administered 3.5% DSS for 7 days. (**b**,**c**) Representative H&E-stained colon sections (scale bars: 50 µm) and histological colitis scores for *Slco2a1*^*fl/fl*^ and *Slco2a1*^*ΔMP*^ mice on day 7 after administration of 3.5% DSS. (**d**) Changes in body weight during the experimental period. *Slco2a1*^*fl/fl*^ (n = 6) and *Slco2a1*^*ΔIEC*^ (n = 6) mice were administered 3.5% DSS for 7 days, and then given normal drinking water for one day. (**e**,**f**) Representative H&E-stained colon sections (scale bars: 50 µm) and histological colitis scores for *Slco2a1*^*fl/fl*^ and *Slco2a1*^*ΔIEC*^ mice on experimental day 8. Data represent the mean ± SEM or median and IQR. Statistical significance was calculated by Student’s *t*-test, Welch’s *t*-test, or Mann-Whitney test (*P < 0.05, or **P < 0.01).

To investigate whether *Slco2a1* deficiency in macrophages is associated with exacerbation of colitis, we developed mice with macrophage-specific deletion of *Slco2a1* (*Lysozyme M-cre;Slco2a1*^*fl/fl*^, hereafter referred to as *Slco2a1*^*ΔMP*^). On only experimental day 4, *Slco2a1*^*ΔMP*^ mice treated with DSS exhibited more severe reductions in body weight than did *Slco2a1*^*fl/fl*^ littermates, although weight loss was not different between the two groups on experimental day 7 (Fig. 4a). Histological inflammatory changes were also significantly increased in *Slco2a1*^*ΔMP*^ mice (Fig. 4b,c). On the other hand, mice with intestinal epithelial cell-specific deletion of *Slco2a1* (*Villin-cre;Slco2a1*^*fl/fl*^, hereafter referred to as *Slco2a1*^*ΔIEC*^) showed less body weight reduction and milder intestinal inflammation than did *Slco2a1*^*fl/fl*^ littermates (Fig. 4d–f).

### Increased PGE_2_ secretion by macrophages in the colon in *Slco2a1*^−/−^ mice might be a potential mechanism of exacerbation of DSS-induced colitis

**Figure 5:**
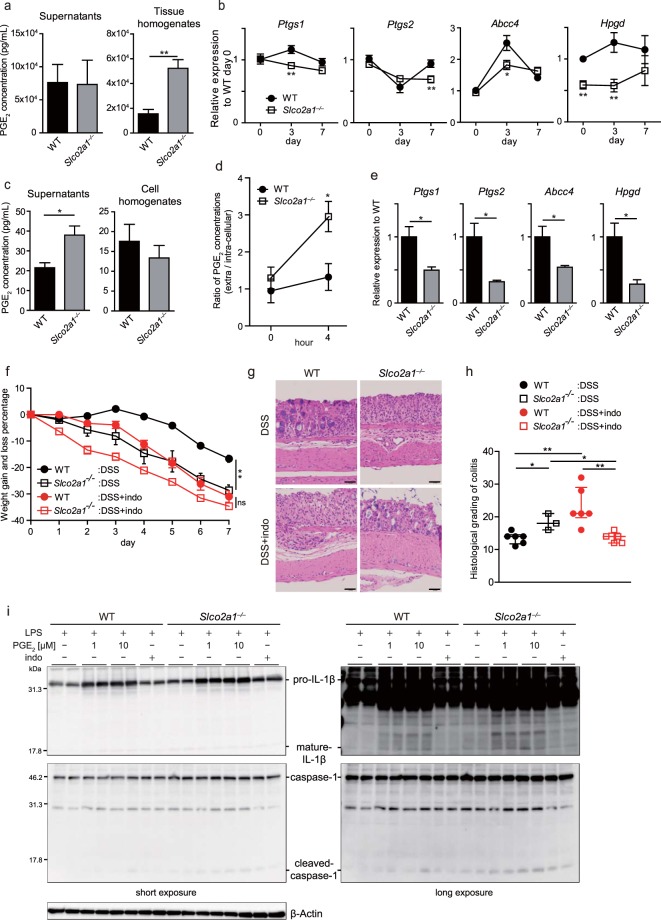
Concentrations of prostaglandin E_2_ (PGE_2_) and expression of PGE_2_-related genes in colon tissues. (**a**) Concentrations of PGE_2_ in supernatants from colon explant cultures and colon tissue homogenates after administration of 3.5% DSS for 3 days were measured by ELISA. (**b**) mRNA levels of *Ptgs 1*, *Ptgs 2*, *Abcc4*, and *Hpgd* relative to those in WT mice on day 0 in colon tissue on days 3 and 7 after administration of 3.5% DSS. (**c**) BMDMs were isolated from WT and *Slco2a1*^−/−^ mice and stimulated with 1 µg/mL of LPS for 4 h. PGE_2_ concentrations in supernatants and cell homogenates were determined by ELISA. (**d**) Extra-to-intracellular PGE_2_ concentration ratios. (**e**) mRNA levels of PGE_2_-related genes in LP macrophages isolated from WT and *Slco2a1*^−/−^ mice treated with 3.5% DSS for 3 days. (**f**) Changes in body weight during the experimental period. WT (n = 6) and *Slco2a1*^−/−^ (n = 6) mice were treated with 3.5% DSS and indomethacin (nonselective cyclooxygenase inhibitor, 1 mg/kg) for 7 days. (**g**,**h**) Representative H&E-stained colon sections (scale bars: 50 µm) and histological colitis scores for WT and *Slco2a1*^−/−^ mice on experimental day 7. (**i**) BMDMs from WT and *Slco2a1*^−/−^ mice were stimulated with LPS (1 μg/mL) and PGE_2_ (1 μM or 10 μM) or indomethacin (10 μM) for 4 h and then analysed IL-1β and caspase-1 by western blotting. Data represent the mean ± SEM or median and IQR. Statistical significance was calculated by Student’s *t*-test, Welch’s *t*-test, or Mann-Whitney test (*P < 0.05, **P < 0.01).

As *Slco2a1* encodes a PGT^6^, we next hypothesized that inflammasome activation in macrophages could be caused by alteration of the PGE_2_ concentration by *Slco2a1* deficiency. In colon tissue of DSS colitis mice on day 7, PGE_2_ concentrations were significantly higher in *Slco2a1*^−/−^ than in WT mice (Fig. 5a), whereas the concentrations did not differ between *Slco2a1*^*ΔIEC*^ and *Slco2a1*^*fl/fl*^ littermates (data not shown). *Ptgs1* and *2* encoding PG synthesizing enzyme COX-1 and 2, respectively, and *Hpgd* encoding PG-inactivating 15-PGDH were downregulated in *Slco2a1*^−/−^ mice (Fig. 5b). Furthermore, PGE_2_ concentrations in colon tissue were significantly higher in *Slco2a1*^*ΔMP*^ administered DSS than in *Slco2a1*^*fl/fl*^ littermates (Supplementary Fig. S6a), although mRNA levels of *Ptgs1*, *Ptgs2*, *Abcc4*, and *Hpgd* were did not differ between both mice (Supplementary Fig. S6b). Therefore, we next explored PGE_2_ production by *Slco2a1*-deficient macrophages, which were identified as key players in DSS colitis exacerbation. PGE_2_ concentrations in extracellular fluids of BMDMs after lipopolysaccharide (LPS) stimulation from *Slco2a1*^−/−^ mice were significantly higher than those in the WT counterparts, although intracellular concentrations did not differ (Fig. 5c,d). mRNA levels of *Ptgs1*, *Ptgs2*, *Abcc4*, and *Hpgd* were significantly decreased in LP macrophages from *Slco2a1*^−/−^ mice with DSS-induced colitis when compared with their WT counterparts (Fig. 5e). The decreased mRNA expression of these genes and the increased extra-to-intracellular PGE_2_ concentration ratio in *Slco2a1*-deficient BMDMs indicated that *Slco2a1* deficiency might contribute to increase in PGE_2_ concentration via inhibition of transport of extracellular PGE_2_ into the cytoplasm.

The PGE_2_/EP2 signalling pathway has been recently reported to boost pro-IL-1β production in BMDMs stimulated with LPS, resulting in mature IL-1β production via NLRP3 activation^25^. Together with our data, this finding suggests that *Slco2a1* deficiency might lead to a decline in PGE_2_ metabolism and thus, a high concentration of PGE_2_ around macrophages, subsequently resulting in exacerbated intestinal inflammation via inflammasome activation in macrophages. In support of this hypothesis, although indomethacin treatment led to considerable bodyweight loss in both WT and *Slco2a1*^−/−^ mice given DSS compared to no treatment (Fig. 5f), *Slco2a1*^−/−^ mice administered DSS and treated with indomethacin exhibited significantly lower histological colitis scores than WT mice administered DSS and indomethacin, and *Slco2a1*^−/−^ mice administered DSS alone (Fig. 5g,h). To assess the mechanistic link between *Slco2a1* deficiency and inflammasome activation via PGE_2_ pathway, BMDMs from WT and *Slco2a1*^−/−^ mice were stimulated with LPS and PGE_2_/COX inhibitor indomethacin. In line with the results *in vivo*, pro-IL-1β and caspase-1 induced by exogenous PGE_2_ were cleaved by LPS stimulation. Those mature IL-1β and cleaved caspase-1 were boosted in BMDMs from *Slco2a1*^−/−^ mice compared to BMDMs from WT mice (Fig. 5i, Supplementary Fig. S7). The upregulated expression of mature IL-1β and cleaved caspase-1 were supressed by the COX inhibitor indomethacin, especially dramatically in BMDMs from *Slco2a1*^−/−^ mice, in support of *in vitro* experiment (Fig. 5i, Supplementary Fig. S7).

## Discussion

**Figure 6:**
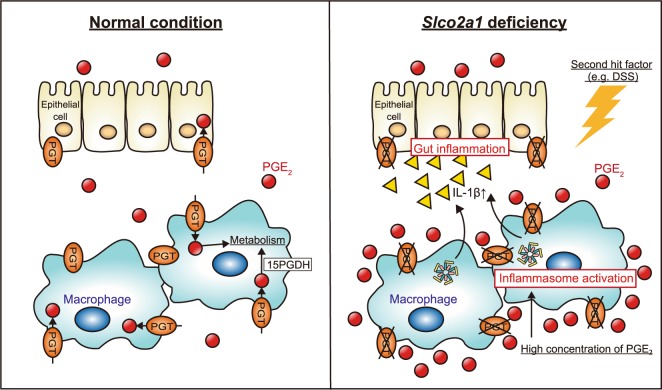
Schematic representation of the possible mechanism of gut inflammation induced by DSS under *Slco2a1*-deficiency. *Slco2a1*-deficiency under DSS stimulation increases the PGE_2_ concentration around macrophages by suppressing PGE_2_ metabolism. The increased PGE_2_ causes NLRP3 inflammasome activation in macrophages, thus exacerbating gut inflammation.

This study showed that *Slco2a1* deficiency led to high concentration of PGE_2_ metabolic pathway, resulting in exacerbated intestinal inflammation via inflammasome activation in macrophages (Fig. 6), which might be a possible mechanism of CEAS.

Various studies have reported that PGs, which are synthesized from arachidonic acid by COXs, and selective prostanoid receptor agonists exert anti-inflammatory and mucosal protective effects in experimental colitis by inhibiting inflammatory cytokines and inducing mucus secretion in intestinal epithelial cells^26,27^. Clinical reports of NSAID-induced intestinal mucosal injury and basic evidence of genetic COX deletion- and COX inhibitor-exacerbated colonic injury in several models of colitis^28–30^ support the beneficial effect of PGE_2_ on intestinal epithelial cells.

On the other hand, studies reporting on roles of molecules related to PGE_2_ metabolism in inflammatory conditions are limited. The PGE_2_ metabolic pathway involves SLCO2A1, which mainly imports PGE_2_ into the cytoplasm, and the metabolic enzyme 15-PGDH, which oxidizes PGE_2_ to 15-keto-PGE_2_^31^. 15-PGDH expression on intestinal epithelial cells is downregulated in patients with active IBD^1^, whereas 15-PGDH reportedly was upregulated by inflammatory cytokines, such as IL-6, in a prostate cancer cell line^32^. These findings indicate that the PGE_2_ metabolic pathway also contributes to an altered response to inflammatory stimuli, depending on the cell type. In fact, *Hpgd* knockout or pharmacologic inhibition of 15-PGDH increased tissue PGE_2_ levels and lowered the susceptibility to DSS colitis in mice^33^.

The present study revealed that germline *Slco2a1*-deficient mice were more susceptible to DSS-induced colitis than WT mice, but did not spontaneously develop enteritis or colitis. This result was opposite to what we expected based on findings in *Hpgd*-knockout mice^33^ and the beneficial effects of PGs and selective prostanoid receptor agonists in the intestine^26,27^. Therefore, we conducted an unbiased microarray assay to identify mechanisms for the exacerbation of experimental colitis in *Slco2a1*-knockout mice. Intriguingly, microarray analysis of colon tissues revealed that inflammasome-related genes, such as *Il1b*, *Il18*, and *P2rx7*, and macrophage-related chemokines, such as *Ccl2* and *Ccl4*, were upregulated in *Slco2a1*-knockout mice administered DSS when compared with WT mice treated with DSS. The cytokine IL-1β has been implicated as a central mediator of the inflammatory processes in patients with IBD and in experimental colitis^34–36^. Inactive pro-IL-1β is cleaved by caspase-1, which is activated within the inflammasome, to generate the mature, active form^37^. The inflammasome is formed by NLRP3, the adaptor protein apoptosis-associated speck-like protein containing a caspase-recruitment domain, and caspase-1^18^. Protein levels of mature IL-1β and cleaved caspase-1 were increased in colon tissues of *Slco2a1*-knockout mice treated with DSS, which led us to hypothesize that NLRP3 inflammasome activation might contribute to the exacerbation of DSS colitis in *Slco2a1*-deficient mice. This hypothesis was proven true by administering mice MCC950, a specific inhibitor of the NLRP3 inflammasome^24,38^, as MCC950 administration reduced the severity of colitis in *Slco2a1*-deficient mice via suppressing the expression of mature IL-1β and cleaved caspase-1.

Several studies have shown that macrophages are the main site of IL-1β production in the colon in IBD patients^39–41^. The NLRP3 inflammasome and subsequent secretion of mature IL-1β by macrophages has been reported as a main mechanism of intestinal inflammation in model mice^19,25^. In line with these findings, LP macrophages and BMDMs isolated from *Slco2a1*-deficient mice showed stronger inflammasome activation than WT mice. Macrophage-specific, but not intestinal epithelial cell-specific *Slco2a1* knockout increased the susceptibility to DSS-induced colitis, partially phenocopying systemic *Slco2a1*-deficient mice, which indicated that Slco2a1 in macrophages might play an important role in maintaining mucosal homeostasis in conditions of injury.

The present study showed that *Slco2a1* deficiency led to an increased PGE_2_ concentration in colon tissues. *In-vitro* experiments using BMDMs corroborated that PGE_2_ concentrations in extracellular fluids of BMDMs from *Slco2a1*-deficient mice were higher than those in their WT counterparts, possibly through suppression of PGE_2_ metabolism. A recent study demonstrated that PGE_2_/EP2 signalling pathway facilitated pro-IL-1β production in BMDMs stimulated with LPS, resulting in mature IL-1β by the NLRP3 inflammasome activation^25^. Increased PGE_2_ production was related with macrophage infiltration in the intestine in mice with DSS-induced colitis^42^. Taking these findings together, it can be considered that the NLRP3 inflammasome in macrophages was autocrinally activated by the elevated concentrations of PGE_2_, leading to exacerbation of colitis in *Slco2a1*-deficient mice. In support of this, exacerbated colitis in mice lacking *Slco2a1* was improved by treatment with the COX inhibitor indomethacin as the concentration of PGE_2_ around macrophages decreased. On the other hand, in WT mice, colitis was worsened by indomethacin administration, which is consistent with finding by Okayama *et al*.^43^. These implied that the severity of colitis would depend on the balance between PGE_2_ mediated anti-inflammatory effect on epithelium and pro-inflammatory effect on activated macrophage. Indomethacin treatment led to considerable body weight loss in both WT and *Slco2a1*-deficient mice treated with DSS when compared to non-treated animals, but this was presumed to be because of mucosal injury of the stomach or small intestine caused by indomethacin^43–45^.

Our observation that increased PGE_2_ levels exacerbated colitis is in contrast to findings in *Hpgd*-deficient mice by Zhang *et al*.^33^, who reported that deletion of 15-PGDH increased tissue PGE_2_ levels and protected mice from experimental colitis. A possible explanation could be the difference in the dominant locations of SLCO2A1 and 15-PGDH expression. Zhang *et al*. concluded that the phenotype of *Hpgd*-deficient mice is due to 15-PGDH deletion in colonic epithelial cells, which are the primary site of 15-PGDH expression in the colon^46^. On the other hand, our study revealed that the phenotype of *Slco2a1*-deficient mice is mainly due to Slco2a1 deletion in macrophages. In fact, intestinal epithelial-specific *Slco2a1*-deficient mice has lower susceptibility to experimental colitis as with the model in the study by Zhang *et al*. Although suppressed PGE_2_ metabolism in macrophages might contribute to the enhanced PGE_2_ concentration, *Slco2a1*^*ΔMP*^ only partially reproduced the systemic knockout phenotype in the present study. As SLCO2A1 is mainly expressed on vascular endothelial cells in the intestinal tracts of mice^47^ and humans^12^, a decrease in the clearance of PGE_2_ from vascular endothelial cells might also contribute to the increase in the PGE_2_ concentration around macrophages in the intestine. Further studies will clarify the role of SLCO2A1 on endothelial cells in intestinal homeostasis.

Although a recent study has demonstrated that *SLCO2A1* function is lost in patients with CEAS^12^, the current study revealed that *Slco2a1*-deficient mice did not spontaneously develop enteritis. This might be because Slco2a1/SLCO2A1 has different functions *in vivo* in mice and humans. Patients with CEAS might have a “second-hit factor” other than lack of SLCO2A1, such as other genetic factors or certain pathogens in the intestinal lumen. Considering the clinical impact, these mice model may not be the best model to analyse CEAS which affects mainly small intestine. Further studies using another enteritis model are needed to clarify the pathogenesis of CEAS.

In summary, *Slco2a1* deficiency increased the PGE_2_ concentration around macrophages, possibly by suppressing PGE_2_ metabolism, resulting in activation of the NLRP3 inflammasome in macrophages and thus exacerbating intestinal inflammation in an experimental colitis model. Our findings shed some light on the pathogenesis of the intestinal inflammation associated with SLCO2A1 and might provide a novel therapeutic target.

## Methods

### Animals

*Slco2a1*^−/−^ and *Slco2a1*^*fl/fl*^ mice on B6 background were obtained from Kanazawa University^14^. *Lysozyme (Lys) M-cre* mice and *Villin (V)-cre* mice were obtained from RIKEN Bioresource Centre (RRID:IMSR_RBRC02302) and Jackson laboratory (B6.Cg-Tg(Vil1-cre)1000Gum/J), respectively. *Slco2a1*^*fl/fl*^ mice were crossed with *LysM-cre* mice and *V-cre* mice to generate mice with specific *Slco2a1* deletion in macrophages (*Slco2a1*^*ΔMP*^)^48^ and intestinal epithelial cells (*Slco2a1*^*ΔIEC*^). Sex- and age-matched WT mice (C57BL/6), obtained from Charles River Japan Inc. (Atsugi, Japan), and sex- and age-matched littermate *LysM-cre*^*−*^;*V-cre*^*−*^;*Slco2a1*^*fl/fl*^ mice were used as controls for *Slco2a1*^−/−^ mice and *Slco2a1*^*ΔMP*^ *Slco2a1*^*ΔIEC*^ mice, respectively. The mice were kept under constant housing conditions (12-h light/dark cycles and 22 ± 1 °C) and had free access to water and standard diet throughout the experimental period. All experiments were approved by the animal care committee of the Osaka City University Graduate School of Medicine (approval number: 16025). All experiments were conducted in accordance with relevant guidelines/regulations.

### Assessment of basal phenotype of *Slco2a1*^−/−^ mice

Body weights of WT and *Slco2a1*^−/−^ mice were recorded weekly until the age of 24 weeks. At the ages of 8, 12, and 24 weeks, gender-matched mice were sacrificed by CO_2_ asphyxiation. Small intestines and colons were immediately collected for evaluation of spontaneous inflammation. The effect of *Slco2a1* knockout is shown in Supplementary Fig. S8.

### Dextran sodium sulphate (DSS)-induced colitis and treatments

Acute colitis was induced by 3.5% (w/v) DSS (molecular weight, 5000; Fujifilm, Osaka, Japan) added to the drinking water for 7 days. Seven- to nine-week-old gender-matched mice were included in each group. Body weight was recorded daily. Mice were sacrificed on day 3 or 7, and the colons were collected. To inhibit the NLRP3 inflammasome *in vivo*, mice received an intraperitoneal injection of 50 mg/kg MCC950 (AdipoGen Life Sciences, San Diego, CA) (or PBS as a control) every other day from one day before to day 7 of DSS administration. To inhibit PGE_2_ production *in vivo*, indomethacin (Nacalai Tesque, Kyoto, Japan) was dissolved in ethanol (10 mg/mL) and was added to the drinking water at 1 mg/kg per day, concurrent with DSS treatment.

### Histopathological scoring of enterocolitis

Tissues were flushed with PBS and fixed 4% paraformaldehyde phosphate buffer solution for 5 days prior to paraffin embedding. Sections (4 μm) were then cut and stained with haematoxylin and eosin (H&E) and alcian blue/periodic acid-Schiff (AB/PAS) using an Alcian Blue-PAS Stain Kit (ScyTek Laboratories, Logan, UT) following the manufacturer’s instructions. Ileum or colon sections were evaluated using a histological scoring system^49^ or histological grading of colitis^50,51^. Goblet and Paneth cells were quantified in at least 100 crypts of each mouse.

### Isolation and stimulation of murine macrophages

LP macrophages were isolated from the mouse colons as described previously^52^. CD11b-positive cells among LP cells were purified by magnetic-activated cell sorting using CD11b MicroBeads (130-049-601; Miltenyi Biotec, Bergisch Gladbach, Germany) following the manufacturer’s instructions.

BMDMs were prepared as described^53,54^. Briefly, bone marrow cells were incubated in Dulbecco’s modified Eagle’s medium (DMEM; Sigma-Aldrich, St. Louis, MO) supplemented with 20% foetal bovine serum (FBS; HyClone, Logan, UT) and 5% antibiotic-antimycotic (Amphotericin B, Penicillin, and Streptomycin; Thermo Fisher Scientific, Waltham, MA) at 37 °C for 4 h. The cells in supernatants were then collected and cultured in 10 mL of DMEM containing 15% L929 cell-conditioned medium, 20% FBS, and 5% antibiotic-antimycotic for 10 days, with a medium change every 2–3 days. BMDMs were cultured in culture dishes or 6-well plates in the absence of FBS. On the following day, the cells were stimulated with 1 µg/mL LPS (*Escherichia coli* 0111:B4, L4391; Sigma-Aldrich) for up to 4 h. 16,16-dimethyl PGE_2_ (dmPGE_2_; Cayman Chemical, Ann Arbor, MI) and indomethacin were added to culture before LPS treatment.

### RNA extraction and quantitative reverse-transcription polymerase chain reaction (RT-qPCR)

Total RNA was extracted from tissues and cells using an ISOGEN II kit (Nippon Gene, Tokyo, Japan) following the manufacturer’s protocol. A High-Capacity RNA-to-cDNA Kit (Thermo Fisher Scientific) was used per the manufacturer’s protocol to convert the total RNA to complementary DNA. qPCRs were run in duplicate using SYBR Select Master Mix (Thermo Fisher Scientific) in an Applied Biosystems 7500 Fast Real-Time PCR system, and data were analysed with the in-built software (Thermo Fisher Scientific). Thermal cycling conditions were as follows: 50 °C for 2 min and 95 °C for 2 min, 40 cycles of denaturation at 95 °C for 3 s and annealing at 60 °C for 30 s. The primers used are listed in Supplementary Table S1. Primers were designed by using Primer-BLAST in the NCBI genome browser. Gene expression was normalized to the expression level of *Hprt* mRNA.

### Western blot analysis

Tissues and isolated cells were homogenized in RIPA lysis buffer (Thermo Fisher Scientific) containing Complete Protease Inhibitor Cocktail (Roche Diagnostics, Mannheim, Germany). Protein content was measured with a Pierce BCA Protein Assay kit (Thermo Fisher Scientific), and proteins were resuspended in Sample Buffer Solution with 3-Mercapto-1,2-Propanediol (4×) (Fujifilm) and heated to 98 °C for 5 min. The proteins were resolved on polyacrylamide gels and transferred to polyvinylidene difluoride membranes. The membranes were incubated with goat anti-IL-1β (1:500; Val118Ser269; R&D Systems, Minneapolis, MN), rabbit anti-caspase-1 (1:200; 14F468; Santa Cruz Biotechnology, Santa Cruz, CA), mouse anti-NLRP3 (1:1,000; Cryo-2; AdipoGen Life Sciences), and mouse anti-β-Actin (1:10,000; AC-15; Sigma-Aldrich) at 4 °C overnight. Proteins were detected using appropriate secondary antibodies conjugated with horseradish peroxidase and enhanced chemiluminescence system (Amersham ECL Prime Western Blotting Detection Reagent; GE Healthcare Life Sciences, Buckinghamshire, UK).

### Colon tissue explant cultures

Colon sections (1 cm) from individual mice were washed with PBS to remove faecal contents and were then cultured in 24-well plates in 500 μL of RPMI 1640 (Nacalai Tesque) containing 2% FBS and antibiotics at 37 °C and in the presence of 5% CO_2_. After a 24-h incubation, the culture medium was collected and centrifuged. The supernatants were transferred to new tubes for enzyme-linked immunosorbent assay (ELISA) of PGE_2_.

### ELISA

Colon tissues and BMDMs were homogenized in 0.1 M phosphate containing 1 mM EDTA and 10 μM indomethacin. The amounts of PGE_2_ in supernatants and tissue or cell lysates were determined by ELISA (PGE_2_ ELISA Kit; Cayman Chemical), according to the manufacturer’s protocol.

### Microarray analysis

Total RNA extracted from colon tissues was purified using an RNAqueous-Micro Kit (Ambion, Austin, TX) for microarray analysis. The quality and quantity of purified RNA were assessed using an Agilent Technologies 2100 Bioanalyzer and NanoDrop spectrophotometer, respectively, and the RNAs were applied to a SurePrint G3 Mouse Gene Expression 8 × 60 K (Agilent, Inc., Santa Clara, CA). Raw data were extracted using Agilent Feature Extraction Software (v11.0.1.1). The raw data for each gene were automatically summarized in the Agilent feature extraction protocol to generate a raw data text file, providing expression data for each gene probed on the array. Data for array probes that had a Flag A were filtered out. Selected gProcessedSignal values were log-transformed and normalized by the quantile method. Hierarchical cluster analysis was performed using complete linkage and Euclidean distance as a measure of similarity. For GSEA^55^, the GSEA v3.0 software (Broad institute, Massachusetts Institute of Technology and Regents of the University of California) was performed with gene set database c5.all.v6.2.symbols.gmt. The number of random sample permutations was set at 1000.

### Statistical analysis

The data are presented as means ± standard errors of the mean (SEMs) or medians plus interquartile ranges (IQRs). Significance of differences among groups was determined by Student’s *t*-test, Welch’s *t*-test, or Mann-Whitney U test. *P* < 0.05 was considered significant.

## Supplementary information

Supplementary Information.

## References

[1] Otani T (2006). Levels of NAD(+)-dependent 15-hydroxyprostaglandin dehydrogenase are reduced in inflammatory bowel disease: evidence for involvement of TNF-alpha. Am. J. Physiol. Gastrointest. Liver Physiol.

[2] Hatazawa R, Ohno R, Tanigami M, Tanaka A, Takeuchi K (2006). Roles of endogenous prostaglandins and cyclooxygenase isozymes in healing of indomethacin-induced small intestinal lesions in rats. J. Pharmacol. Exp. Ther..

[3] Yao C, Narumiya S (2019). Prostaglandin-cytokine crosstalk in chronic inflammation. Br. J. Pharmacol..

[4] Menter DG, Dubois RN (2012). Prostaglandins in cancer cell adhesion, migration, and invasion. Int. J. Cell Biol..

[5] Breyer RM, Bagdassarian CK, Myers SA, Breyer MD (2001). Prostanoid receptors: subtypes and signaling. Annu. Rev. Pharmacol. Toxicol..

[6] Kanai N (1995). Identification and characterization of a prostaglandin transporter. Sci..

[7] Schuster VL (1998). Molecular mechanisms of prostaglandin transport. Annu. Rev. Physiol..

[8] Bao Y (2002). Prostaglandin transporter PGT is expressed in cell types that synthesize and release prostanoids. Am. J. Physiol. Ren. Physiol.

[9] Nomura T, Lu R, Pucci ML, Schuster VL (2004). The two-step model of prostaglandin signal termination: *in vitro* reconstitution with the prostaglandin transporter and prostaglandin 15 dehydrogenase. Mol. Pharmacol..

[10] Matsumoto T (2004). Non-specific multiple ulcers of the small intestine unrelated to non-steroidal anti-inflammatory drugs. J. Clin. Pathol..

[11] Matsumoto T, Iida M, Matsui T, Yao T (2007). Chronic nonspecific multiple ulcers of the small intestine: a proposal of the entity from Japanese gastroenterologists to Western enteroscopists. Gastrointest. Endosc..

[12] Umeno J (2015). A Hereditary Enteropathy Caused by Mutations in the SLCO2A1 Gene, Encoding a Prostaglandin Transporter. PLoS Genet..

[13] Matsuno Y (2019). Measurement of prostaglandin metabolites is useful in diagnosis of small bowel ulcerations. World J. Gastroenterol..

[14] Nakanishi T (2015). Prostaglandin Transporter (PGT/SLCO2A1) Protects the Lung from Bleomycin-Induced Fibrosis. PLoS One.

[15] Dionne S, Hiscott J, D’Agata I, Duhaime A, Seidman EG (1997). Quantitative PCR analysis of TNF-alpha and IL-1 beta mRNA levels in pediatric IBD mucosal biopsies. Dig. Dis. Sci..

[16] McCauley HA, Guasch G (2015). Three cheers for the goblet cell: maintaining homeostasis in mucosal epithelia. Trends Mol. Med..

[17] Clevers HC, Bevins CL (2013). Paneth cells: maestros of the small intestinal crypts. Annu. Rev. Physiol..

[18] Martinon F, Tschopp J (2004). Inflammatory caspases: linking an intracellular innate immune system to autoinflammatory diseases. Cell.

[19] Bauer C (2010). Colitis induced in mice with dextran sulfate sodium (DSS) is mediated by the NLRP3 inflammasome. Gut.

[20] Bauer C, Duewell P, Lehr HA, Endres S, Schnurr M (2012). Protective and aggravating effects of Nlrp3 inflammasome activation in IBD models: influence of genetic and environmental factors. Dig. Dis..

[21] Higashimori A (2016). Mechanisms of NLRP3 inflammasome activation and its role in NSAID-induced enteropathy. Mucosal Immunol..

[22] Opipari A, Franchi L (2015). Role of inflammasomes in intestinal inflammation and Crohn’s disease. Inflamm. Bowel Dis..

[23] Lehle AS (2019). Intestinal Inflammation and Dysregulated Immunity in Patients With Inherited Caspase-8 Deficiency. Gastroenterology.

[24] Coll RC (2015). A small-molecule inhibitor of the NLRP3 inflammasome for the treatment of inflammatory diseases. Nat. Med..

[25] Zaslona Z (2017). The Induction of Pro-IL-1beta by Lipopolysaccharide Requires Endogenous Prostaglandin E2 Production. J. Immunol..

[26] Tessner TG, Cohn SM, Schloemann S, Stenson WF (1998). Prostaglandins prevent decreased epithelial cell proliferation associated with dextran sodium sulfate injury in mice. Gastroenterology.

[27] Nitta M (2002). Expression of the EP4 prostaglandin E2 receptor subtype with rat dextran sodium sulphate colitis: colitis suppression by a selective agonist, ONO-AE1-329. Scand. J. Immunol..

[28] Higuchi K (2009). Present status and strategy of NSAIDs-induced small bowel injury. J. Gastroenterol..

[29] Morteau O (2000). Impaired mucosal defense to acute colonic injury in mice lacking cyclooxygenase-1 or cyclooxygenase-2. J. Clin. Invest..

[30] Ishikawa TO, Oshima M, Herschman HR (2011). Cox-2 deletion in myeloid and endothelial cells, but not in epithelial cells, exacerbates murine colitis. Carcinogenesis.

[31] Kochel TJ, Fulton AM (2015). Multiple drug resistance-associated protein 4 (MRP4), prostaglandin transporter (PGT), and 15-hydroxyprostaglandin dehydrogenase (15-PGDH) as determinants of PGE2 levels in cancer. Prostaglandins Other Lipid Mediat..

[32] Tong M, Tai HH (2004). Synergistic induction of the nicotinamide adenine dinucleotide-linked 15-hydroxyprostaglandin dehydrogenase by an androgen and interleukin-6 or forskolin in human prostate cancer cells. Endocrinol..

[33] Zhang Y (2015). TISSUE REGENERATION. Inhibition of the prostaglandin-degrading enzyme 15-PGDH potentiates tissue regeneration. Sci..

[34] Kwon KH, Murakami A, Hayashi R, Ohigashi H (2005). Interleukin-1beta targets interleukin-6 in progressing dextran sulfate sodium-induced experimental colitis. Biochem. Biophys. Res. Commun..

[35] Sartor RB (1994). Cytokines in intestinal inflammation: pathophysiological and clinical considerations. Gastroenterology.

[36] Ishiguro Y (1999). Mucosal proinflammatory cytokine production correlates with endoscopic activity of ulcerative colitis. J. Gastroenterol..

[37] Schroder K, Tschopp J (2010). The inflammasomes. Cell.

[38] Primiano MJ (2016). Efficacy and Pharmacology of the NLRP3 Inflammasome Inhibitor CP-456,773 (CRID3) in Murine Models of Dermal and Pulmonary Inflammation. J. Immunol..

[39] Youngman KR (1993). Localization of intestinal interleukin 1 activity and protein and gene expression to lamina propria cells. Gastroenterology.

[40] McAlindon ME, Hawkey CJ, Mahida YR (1998). Expression of interleukin 1 beta and interleukin 1 beta converting enzyme by intestinal macrophages in health and inflammatory bowel disease. Gut.

[41] Ng SC (2011). Relationship between human intestinal dendritic cells, gut microbiota, and disease activity in Crohn’s disease. Inflamm. Bowel Dis..

[42] Yamashita S (1993). Studies on changes of colonic mucosal PGE2 levels and tissue localization in experimental colitis. Gastroenterol. Jpn..

[43] Okayama M (2007). Aggravation by selective COX-1 and COX-2 inhibitors of dextran sulfate sodium (DSS)-induced colon lesions in rats. Dig. Dis. Sci..

[44] Wallace JL (1997). Nonsteroidal anti-inflammatory drugs and gastroenteropathy: the second hundred years. Gastroenterology.

[45] Ilahi M, Khan J, Inayat Q, Abidi TS (2006). Histological changes in parts of foregut of rat after indomethacin administration. J. Ayub Med. Coll. Abbottabad.

[46] Okayasu I (1990). A novel method in the induction of reliable experimental acute and chronic ulcerative colitis in mice. Gastroenterology.

[47] Nakanishi T (2017). A novel role for OATP2A1/SLCO2A1 in a murine model of colon cancer. Sci. Rep..

[48] Nakamura Y (2018). Prostaglandin Transporter OATP2A1/SLCO2A1 Is Essential for Body Temperature Regulation during Fever. J. Neurosci..

[49] Collett A (2008). Early molecular and functional changes in colonic epithelium that precede increased gut permeability during colitis development in mdr1a(−/−) mice. Inflamm. Bowel Dis..

[50] Burns RC (2001). Antibody blockade of ICAM-1 and VCAM-1 ameliorates inflammation in the SAMP-1/Yit adoptive transfer model of Crohn’s disease in mice. Gastroenterology.

[51] Dieleman LA (1998). Chronic experimental colitis induced by dextran sulphate sodium (DSS) is characterized by Th1 and Th2 cytokines. Clin. Exp. Immunol..

[52] Harusato A, Geem D, Denning TL (2016). Macrophage Isolation from the Mouse Small and Large Intestine. Methods Mol. Biol..

[53] Zhang X, Goncalves R, Mosser DM (2008). The isolation and characterization of murine macrophages. Curr. Protoc. Immunol..

[54] Kim S, Joo YE (2011). Theaflavin Inhibits LPS-Induced IL-6, MCP-1, and ICAM-1 Expression in Bone Marrow-Derived Macrophages Through the Blockade of NF-kappaB and MAPK Signaling Pathways. Chonnam Med. J..

[55] Subramanian A (2005). Gene set enrichment analysis: a knowledge-based approach for interpreting genome-wide expression profiles. Proc. Natl Acad. Sci. USA.

