# Supplementary material for: Slco2a1 deficiency exacerbates experimental colitis via inflammasome activation in macrophages: a possible mechanism of chronic enteropathy associated with SLCO2A1 gene
Source: Sci Rep. 2020 Mar 17;10:4883. doi: 10.1038/s41598-020-61775-9 (PMC7078201; doi:10.1038/s41598-020-61775-9)
Supplement: Supplementary file 1 — Supplementary Information. [file 41598_2020_61775_MOESM1_ESM.pdf]

Supplementary information

**_Slco2a1_ deficiency exacerbates experimental colitis via inflammasome activation in macrophages: a possible mechanism of chronic enteropathy associated with _SLCO2A1_ gene**

Rieko Nakata[1], Yoshinobu Nakamura[2], Shuhei Hosomi[1*], Hiroaki Okuda[1], Yu Nishida[1], Naoko Sugita[1], Shigehiro Itani[1], Yuji Nadatani[1], Koji Otani[1], Fumio Tanaka[1], Noriko Kamata[1], Koichi Taira[1], Yasuaki Nagami[1], Tetsuya Tanigawa[1], Toshio Watanabe[1], Hirokazu Yamagami[1], Takeo Nakanishi[3], Yasuhiro Fujiwara[1]

[1] Department of Gastroenterology, Osaka City University Graduate School of Medicine, Osaka, Japan

[2] Department of Pharmacy, Shiga University of Medical Science Hospital, Shiga, Japan

[3] Faculty of Pharmacy, Takasaki University of Health and Welfare, Gunma, Japan

***Corresponding author:**

Supplementary Fig. 1

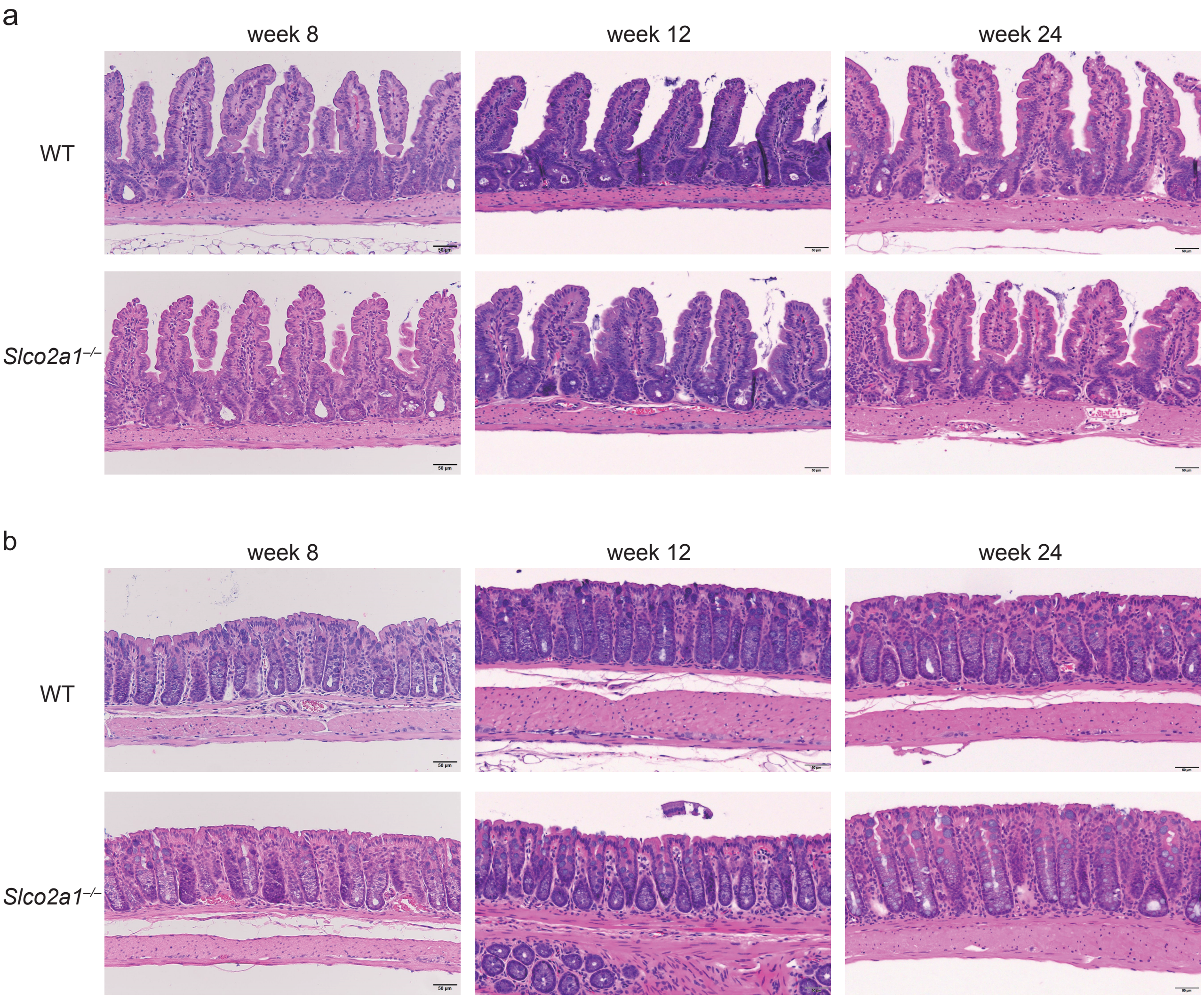


**Supplementary Fig. 1. Representative H&E staining images of WT and *Slco2a1*$^{-/-}$ mice.**

(a, b) Representative H&E-stained ileum (a) and colon (b) sections from WT and *Slco2a1*$^{-/-}$ mice at week 8, 12, and 24 (scale bars: 50 µm).

Supplementary Fig. 2

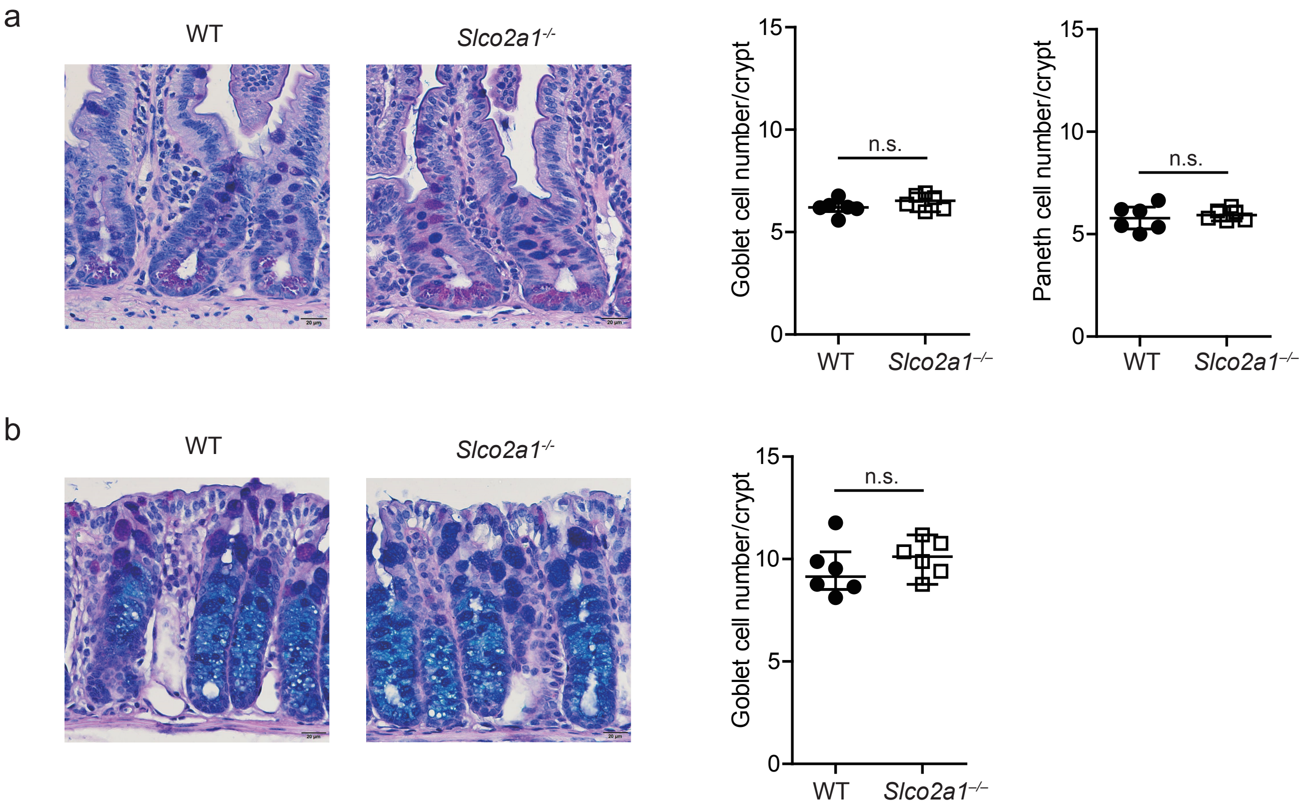


**Supplementary Fig. 2. *Slco2a1* deficiency did not change the number of goblet and Paneth cells in ileum and colon.**

(a, b) Representative Alcian blue/Periodic acid-Schiff staining of ileum and colon sections (scale bars: 20 µm) and quantification of the number of goblet and Paneth cells per crypt from 8-week-old WT (n = 6) and *Slco2a1*$^{-/-}$ (n = 6) mice. Data represent median and interquartile range (IQR). Statistical significance was calculated by Mann-Whitney test.

Supplementary Fig. S3

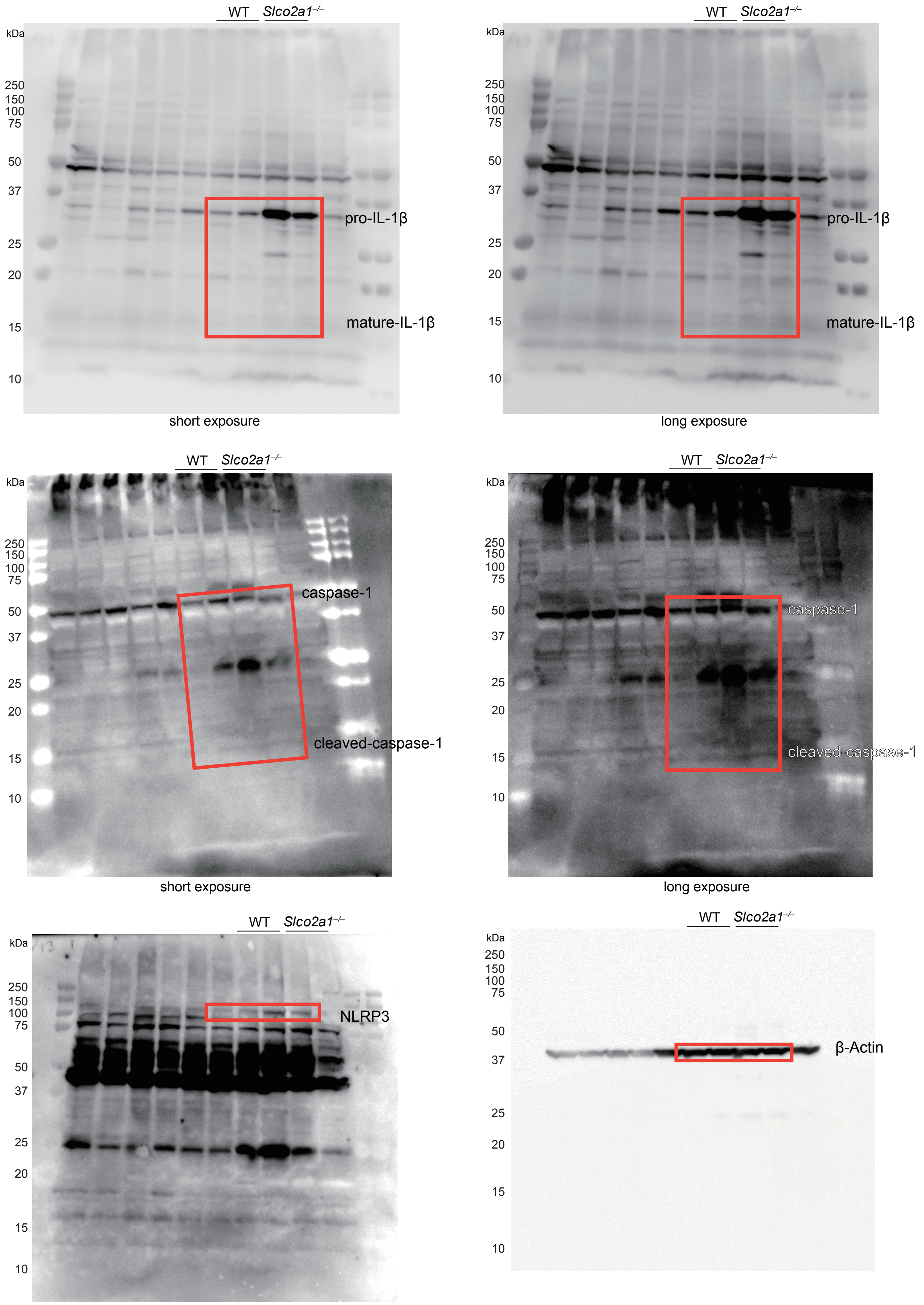


**Supplementary Fig. S3. Full-length Western blot images for Fig. 3c.**

Supplementary Fig. S4

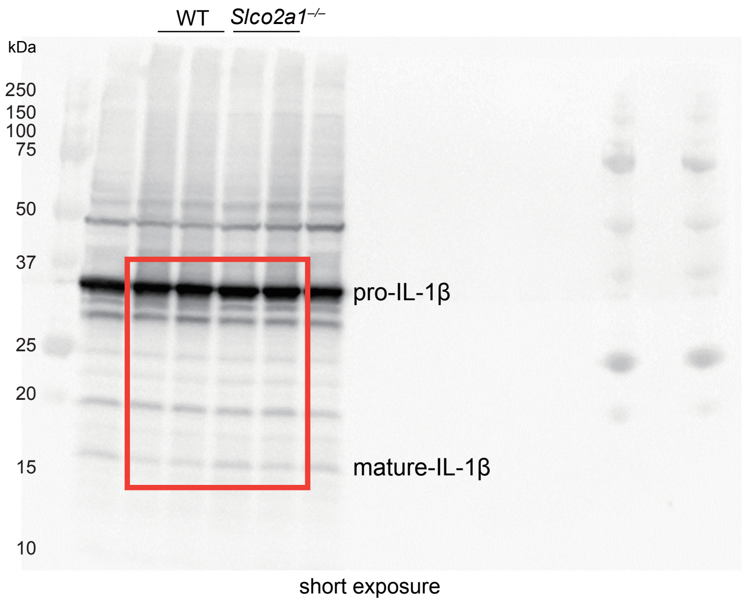


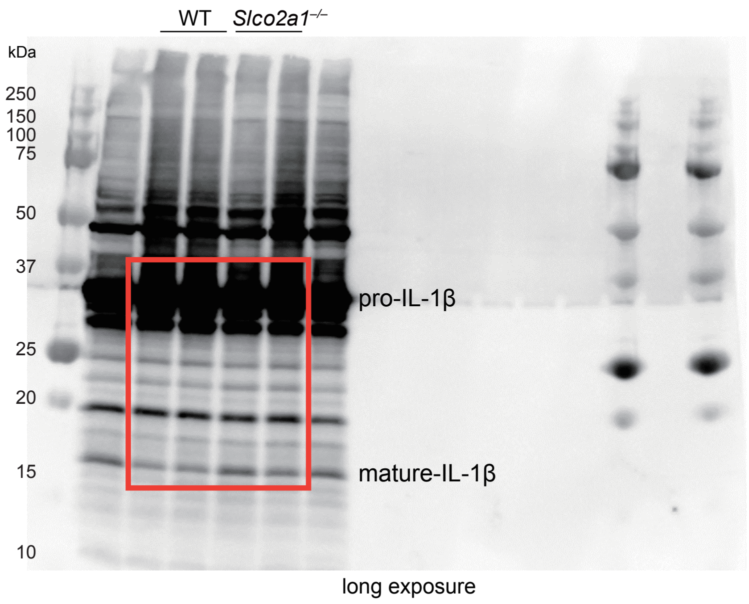


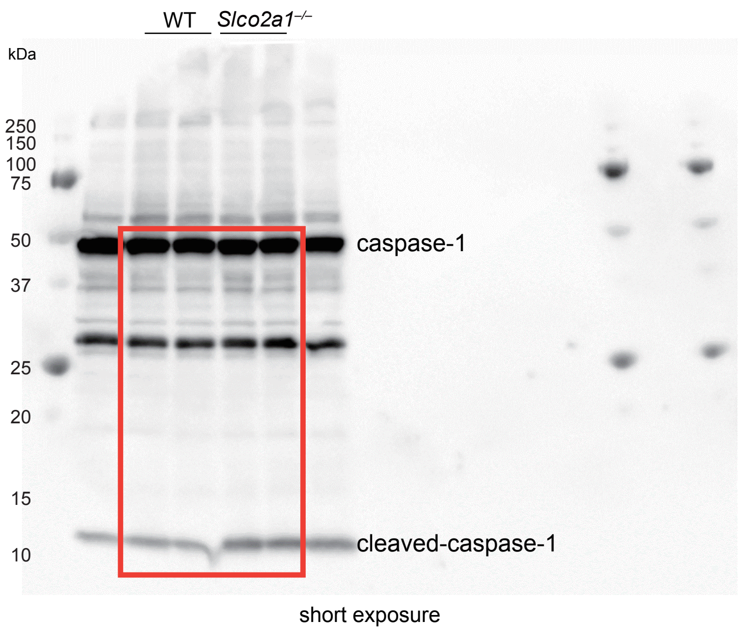


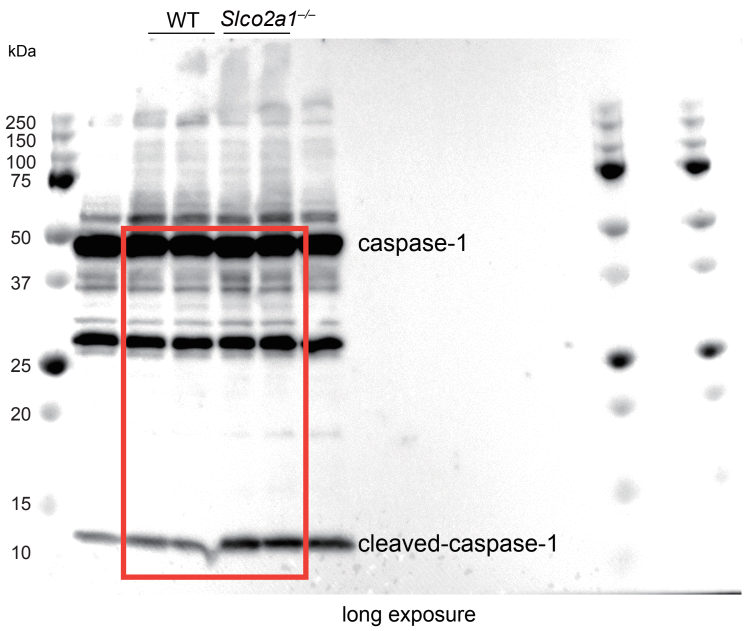


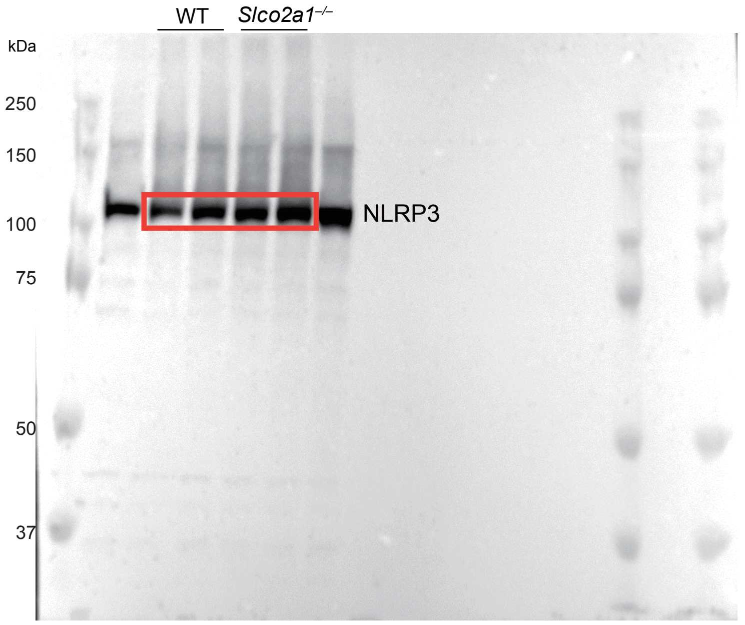


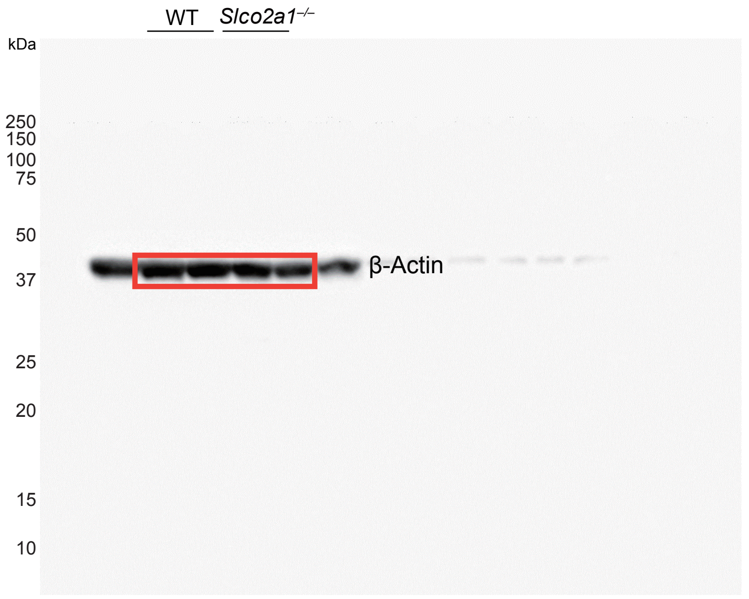


**Supplementary Fig. S4. Full-length Western blot images for Fig. 3e.**

Supplementary Fig. S5

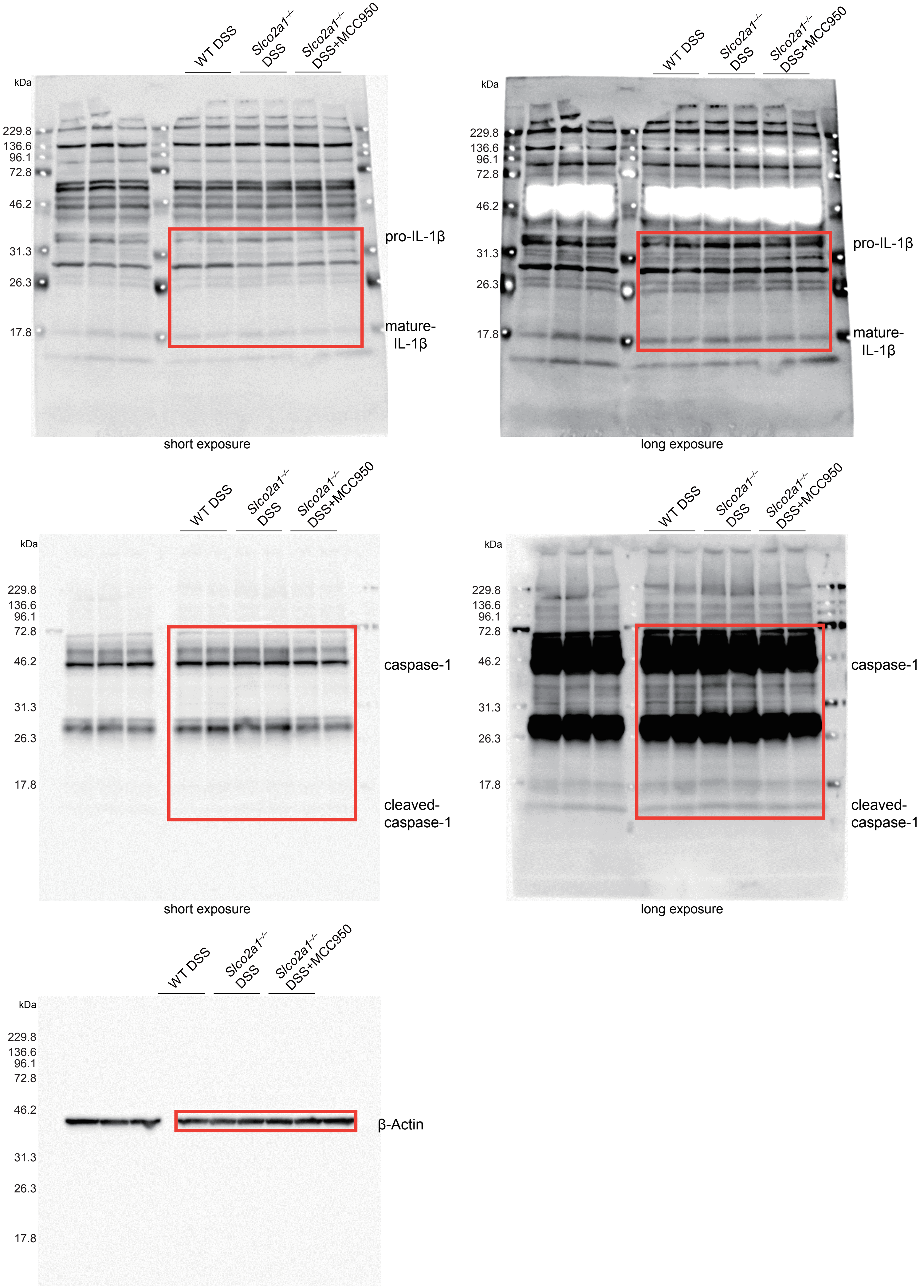


**Supplementary Fig. S5. Full-length Western blot images for Fig. 3j.**

Supplementary Fig. 6

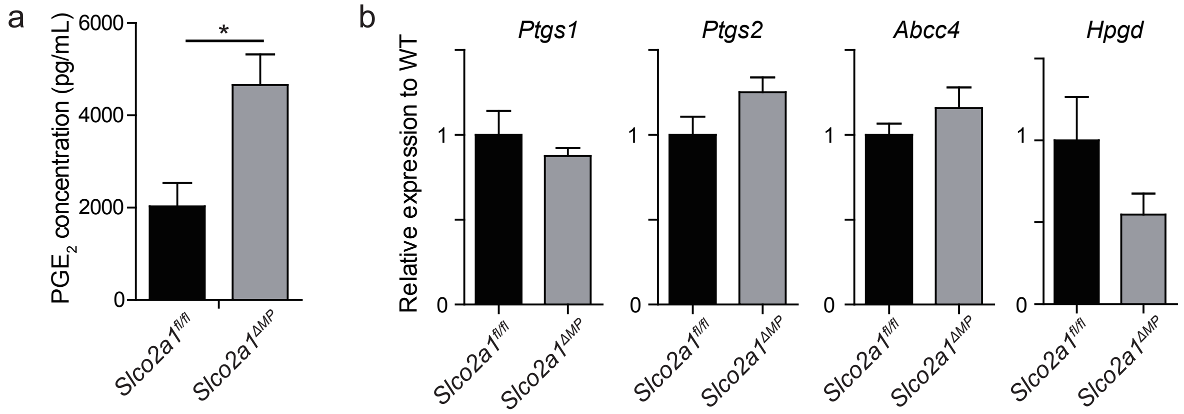


**Supplementary Fig. 6. Concentrations of prostaglandin E2 (PGE2) and expression of PGE2-related genes in colon tissues from *Slco2a1$^{fl/fl}$* and *Slco2a1$^{\Delta MP}$* mice.**

(a) Concentrations of PGE2 in colon tissue homogenates after administration of 3.5% DSS were measured by ELISA. (b) mRNA levels of *Ptgs 1*, *Ptgs 2*, *Abcc4*, and *Hpgd* relative to those in *Slco2a1$^{fl/fl}$* mice administred 3.5% DSS in colon tissue. Data represent the mean ± SEM. Statistical significance was calculated by Student' t-test (**P* < 0.05).

Supplementary Fig. S7

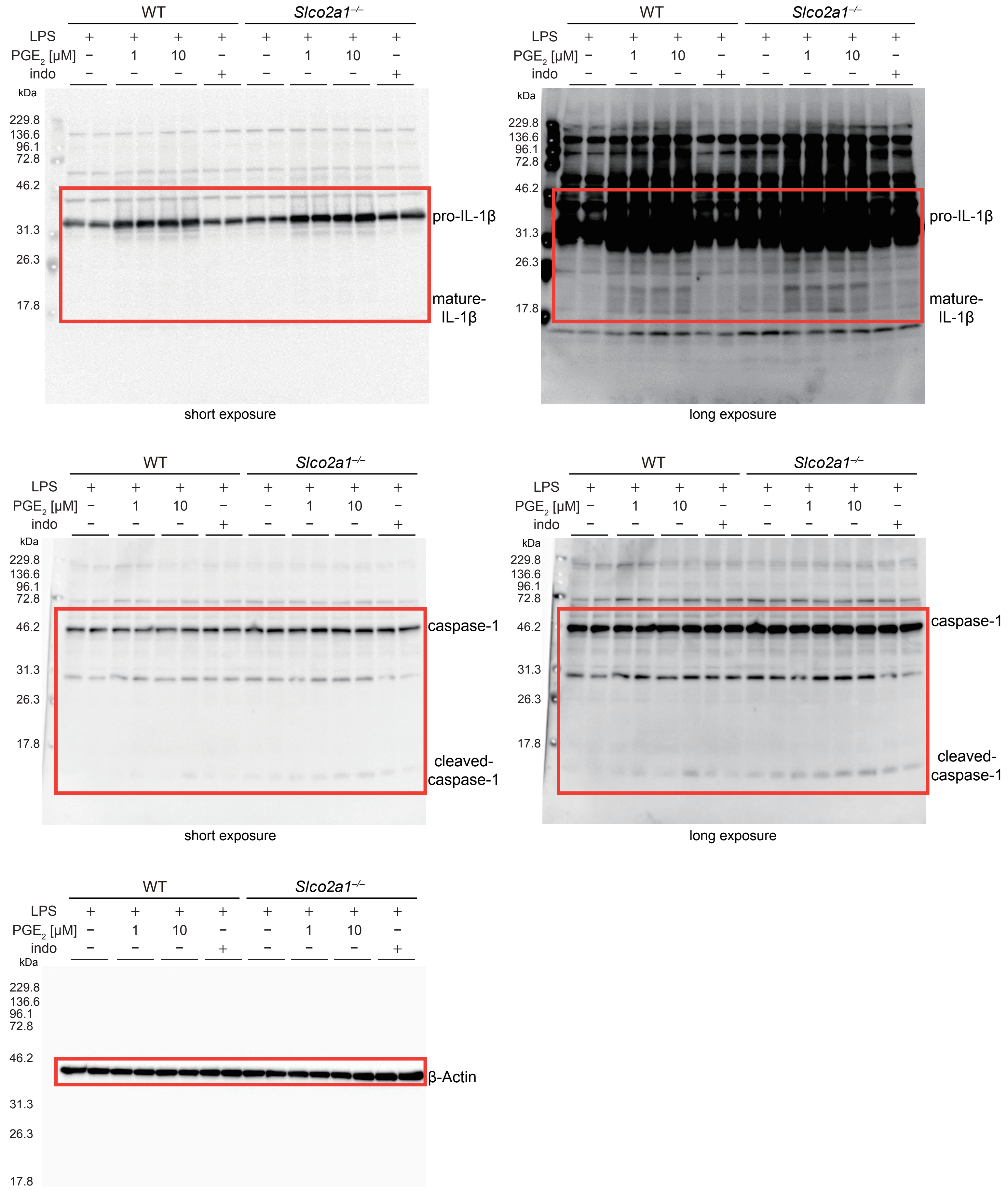


**Supplementary Fig. S7. Full-length Western blot images for Fig. 5i.**

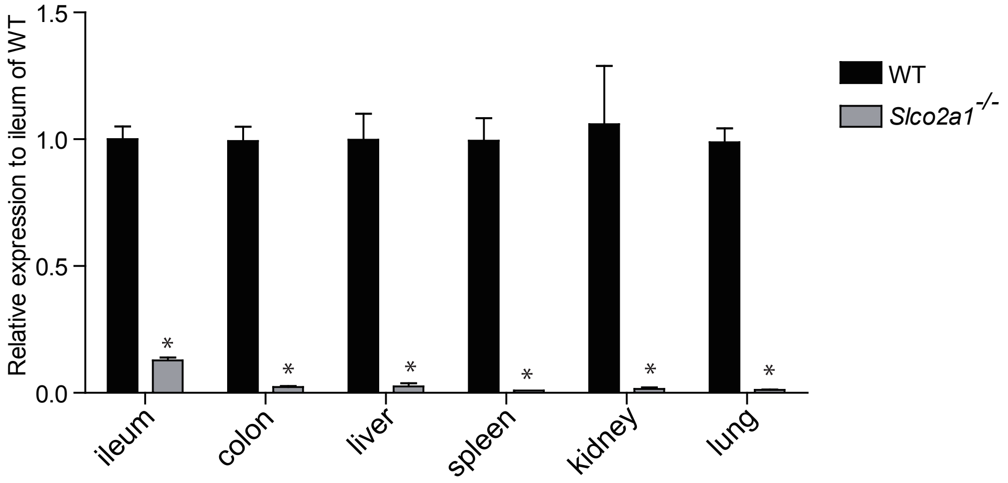


**Supplementary Fig. 8. mRNA expression of *Slco2a1* in the tissue of ileum, colon, liver, spleen, kidney, and lung from WT and *Slco2a1*$^{-/-}$ mice.**

Data represent the mean ± SEM. Statistical significance was calculated by Student' t-test and Welch' s-t test (*$P$ < 0.01).

**Supplementary Table 1. PCR primers**

| *Gene name* | Primers | Sequences (5'–3') |
|---|---|---|
| *Il1b* | Forward | GCCACCTTTTGACAGTGATGA |
| | Reverse | ATGTGCTGCTGCGAGATTTG |
| *Tnf* | Forward | CCCTCCTGGCCAACGGCATG |
| | Reverse | TCGGGGCAGCCTTGTCCCTT |
| *Il6* | Forward | TGCAAGAGACTTCCATCCAGTTGCC |
| | Reverse | TGTGAAGTAGGGAAGGCCGTGGT |
| *Nlrp3* | Forward | CCACATCTGATTGTGTTAATGGCT |
| | Reverse | GGGCTTAGGTCCACACAGAA |
| *Pycard* | Forward | CTGCAGATGGACGCCATAGA |
| | Reverse | AGCTCCAGACTCTTCTTTAGTCG |
| *Casp1* | Forward | GAGCTTCAATCAGCTCCATCAG |
| | Reverse | AACTTGAGGGTCCCAGTCAG |
| *Il18* | Forward | AGTTTACAAGCATCCAGGCACAG |
| | Reverse | TCCAGAAAGCATGGAACCACA |
| *Ptegr1* | Forward | CCACCTCTAGCCGGAAATCT |
| | Reverse | GTGTTATGGCTGGGGGTTTC |
| *Ptegr2* | Forward | TCTGAAGACGTCCTCCACTC |
| | Reverse | AACCCTGGTCGGTTTGATGT |
| *Abcc4* | Forward | GTGCACACCGAGGTGAAAC |
| | Reverse | TTGAGCCACCAGAAGAACAC |
| *Hpgd* | Forward | ATGTCATTTGCCCAGGCTTTG |
| | Reverse | GGCAATGGTTGATGGGTGTAAAA |
| *Hprt* | Forward | AGCGTCGTGATTAGCGATGA |
| | Reverse | GCAAGTCTTTCAGTCCTGTCC |
